# Effectiveness of adjunct telephone-based postnatal care on maternal and infant illness in the Greater Accra Region, Ghana: a randomized controlled trial

**DOI:** 10.1186/s12884-022-05138-4

**Published:** 2022-10-29

**Authors:** Donne Kofi Ameme, Patricia Akweongo, Edwin Andrew Afari, Charles Lwanga Noora, Richard Anthony, Ernest Kenu

**Affiliations:** 1 Department of Epidemiology and Disease Control, School of Public Health, College of Health Sciences, University of Ghana, Legon, Accra, Ghana; 2 Ghana Field Epidemiology and Laboratory Training Programme, School of Public Health, University of Ghana, Legon, Accra, Ghana; 3 Department of Health Policy Planning and Management, School of Public Health, College of Health Sciences, University of Ghana Legon, Accra, Ghana; 4 Department of Medicine, Tema General Hospital, Tema, Ghana

**Keywords:** Postnatal, Follow-up, Telephone, Effectiveness, Maternal, Newborn, Infant, Health outcomes

## Abstract

**Introduction:**

Globally, postnatal care (PNC) is fraught with challenges. Despite high PNC coverages in Ghana’s Greater Accra Region (GAR), maternal and newborn health outcomes are of great concern. In 2017, neonatal and post-neonatal mortality rates in GAR were 19 and 13 per 1000 live births respectively despite PNC coverages of 93% for at least one PNC and 87.5% for PNC within 48 hours post-delivery. Telephone follow-up has been used to improve health outcomes in some settings, however, its usefulness in improving maternal and infant health during the postnatal period is not well known in Ghana. We assessed effectiveness of telephone-based PNC on infant and maternal illness in selected hospitals in GAR.

**Methods:**

An open-label, assessor-blinded, parallel-group, two-arm superiority randomized controlled trial with 1:1 allocation ratio was conducted from September 2020 to March 2021. Mother-baby pairs in intervention arm, in addition to usual PNC, received midwife-led telephone counselling within 48 hours post-discharge plus telephone access to midwife during postnatal period. In control arm, only usual PNC was provided. Descriptive and inferential data analyses were conducted to generate frequencies, relative frequencies, risk ratios and 95% confidence intervals. Primary analysis was by intention-to-treat (ITT), complemented by per-protocol (PP) analysis.

**Results:**

Of 608 mother-baby pairs assessed for eligibility, 400 (65.8%) were enrolled. During 3 months follow-up, proportion of infants who fell ill was 62.5% in intervention arm and 77.5% in control arm (*p* = 0.001). Maternal illness occurred in 27.5% of intervention and 38.5% of control participants (*p* = 0.02). Risk of infant illness was 20% less in intervention than control arm in both ITT analysis [RR = 0.8 (95%CI = 0.71–0.92] and PP analysis [RR = 0.8 (95%CI = 0.67–0.89)]. Compared to controls, risk of maternal illness in intervention arm was 30% lower in both ITT [RR = 0.7 (95%CI = 0.54–95.00)] and PP analysis [RR = 0.7 (95%CI = 0.51–0.94)].

**Conclusion:**

Telephone-based PNC significantly reduced risk of maternal and infant illness within first 3 months after delivery. This intervention merits consideration as a tool for adoption and scale up to improve infant and maternal health.

**Trial registration:**

This trial was retrospectively registered with the International Standard Randomized Controlled Trial Number (ISRCTN) Registry with number ISRCTN46905855 on 09/04/2021.

## Introduction

The postnatal period, which begins immediately after childbirth and extends up to 6 weeks (42 days) after birth remains a critical phase in the lives of mothers and their newborn babies [1]. Majority of maternal and infant deaths occur in this vulnerable period. The postnatal period thus provides a critical window of opportunity for delivering lifesaving interventions to both mother and newborn [2]. This is achieved through effective postnatal care (PNC)-care provided for the mothers and newborns from birth of the babies and for the first 6 weeks of life, with the aim of providing optimum health for mother and the newborn babies [1]. Postnatal care is of immense benefit to maternal and newborn health as it creates the opportunity for identification and management of post-natal complications and provide relevant information to mothers on maternal and newborn care during the post-natal period [3, 4].

The World Health Organization (WHO) guidelines on PNC recommends a total of four postnatal visits for every mother and baby as follows: first day (24 hours), third day (48–72 hours), between days 7 and 14, and 6 weeks after birth for health facility deliveries. The first and second postnatal contacts, which are critical for assessing the mother and newborn for complications and teaching the mother essential newborn and selfcare practices, are expected to take place in the health facility prior to discharge, for deliveries occurring in the health facility [5–7]. Ghana’s PNC schedule consists of at least three contacts for the mother and baby (predischarge care within first 48 hours after delivery, second visit on day 6 or 7 after delivery and last postnatal visit at 6 weeks) [8].

Despite the high PNC coverages in Ghana’s Greater Accra Region, maternal and newborn health outcomes in the region are of great concern, casting doubt on the effectiveness of postnatal interventions. In 2017, 93% of newborns and their mothers received at least one PNC and 87.5% received PNC within the first 48 hours after delivery in the region [9]. In spite of that, neonatal and post-neonatal mortality rates were 19 and 13 per 1000 live births respectively.

Aside being largely client-initiated and heavily dependent on effort of the mothers to seek care [10], usual health facility-based PNC in its current form is over reliant on pre-discharge group counselling and limited to specific scheduled intervals post discharge [1, 11]. These deprive nursing mothers of tailor-made solutions to their problems prior to discharge and uninterrupted access to care while at home. In other instances, particularly in resource-limited settings, mothers are discharged too early. This implies that pre-discharge postnatal contact and counselling, which may be the only postnatal contact received by some mothers, is given too early at the time the mothers are exhausted from childbirth and therefore unable to retain or recall information [11]. Premature discharge of mothers also makes the postpartum length of stay in health facilities too short, leaving insufficient time to recognize and manage complications [12]. Maintaining a continuous linkage with care thus becomes essential for the mothers. In Ghana, however, communication between health workers and mothers throughout the postnatal checks has been observed to be poor [13], with only 23% of nursing mothers surveyed from selected health facilities in the Greater Accra Region reportedly having access to telephone contacts of health workers to clarify concerns during the postnatal period [11].

Though home-based postnatal visit has been used elsewhere to improve maternal and infant health outcomes [3, 14–17], it is relatively expensive and very difficult to sustain on a large-scale under routine conditions. It does not also guarantee provision of continuously accessible care and information for the mothers and their newborns unlike use of telephone which has produced successful results in some settings [18, 19]. Ghana has a high mobile phone penetration of 131.38% with the Greater Accra Region being the region with the highest mobile phone ownership of 73.7% [20, 21] However, the question that remains unanswered is, whether incorporating telephone follow-up into usual PNC, substantially reduces illness among infants and their mothers in low-resource urban settings. We evaluated the effect of telephone follow-up comprising telephone counselling initiated within 48 hours after discharge and telephone access to a midwife throughout the postnatal period as an adjunct to usual PNC on infant and maternal illness in selected hospitals in the Greater Accra Region of Ghana.

## Methods

### Study design

The study was a two-arm, parallel-group, open-label randomized controlled trial conducted across two hospitals, with blinding of the outcome assessors to compare the effectiveness of telephone-based PNC plus usual hospital-based PNC with usual hospital-based PNC alone on infant and maternal illness. The study was designed to answer the question: Among infants and their mothers, how does complementary telephone-based health education initiated within 48 hours after discharge plus telephone access of mother to a dedicated health worker throughout the postnatal period compared with usual PNC alone affect infant and maternal health within the first 3 months after delivery?

### Study area

**Fig. 1:**
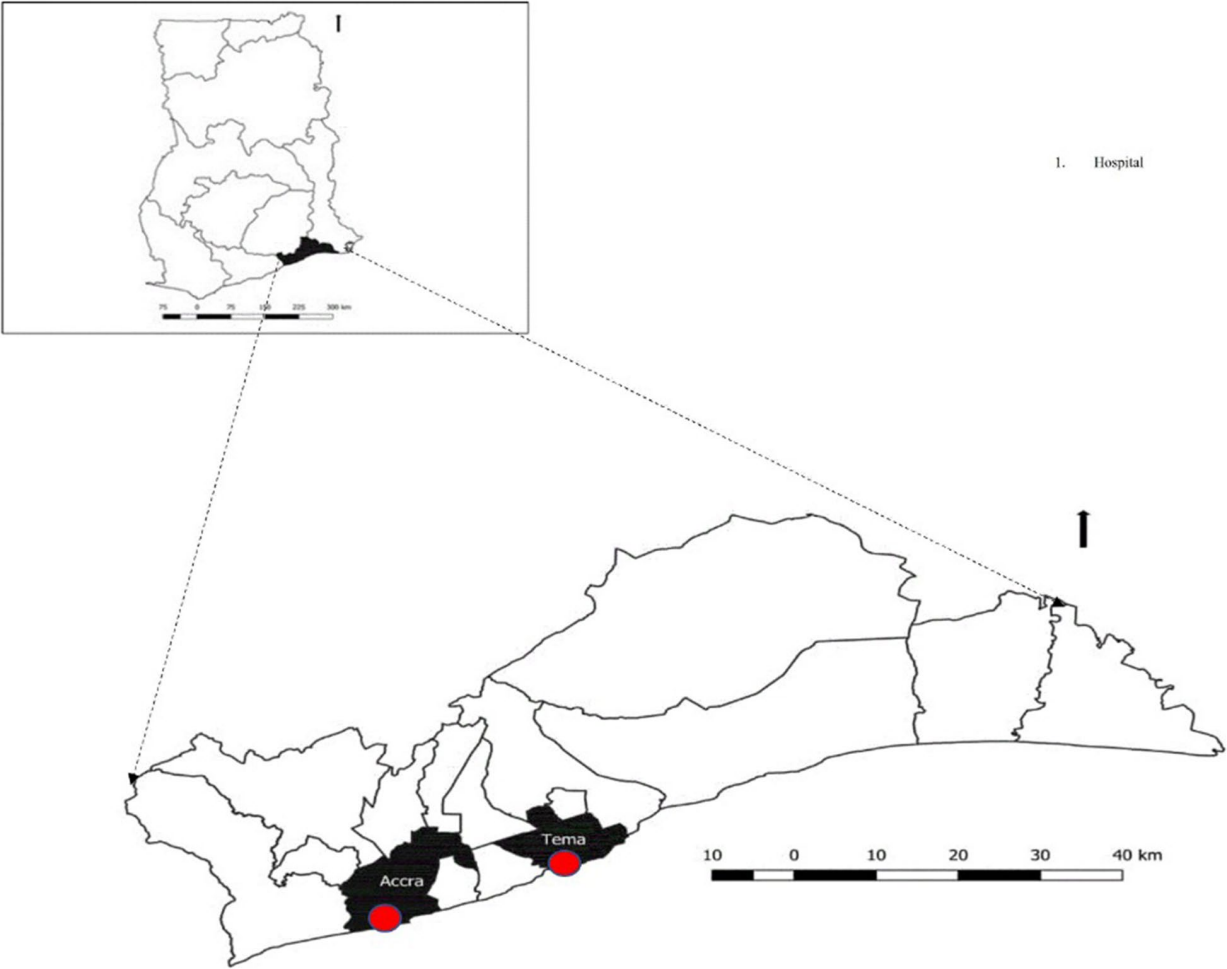
Map of Greater Accra Region showing Accra and Tema Metropolitan Areas and the study sites

The trial was conducted at the Greater Accra Regional and Tema General Hospitals in the Greater Accra Region located in the southeastern part of Ghana (Fig. 1). The Greater Accra Regional Hospital and the Tema General Hospital are two largest hospitals and referral centres in the Accra and Tema Metropolitan Areas. These two metropolises were chosen because they are the largest and most cosmopolitan of the 29 Metropolitan, Municipal and District Assemblies (MMDAs) in the Greater Accra Region. The Greater Accra regional capital city, Accra, serves as both the national and regional capital and is about 30 km from Tema, the capital city of Tema Metropolitan Area.

The Greater Accra Regional Hospital has a 620-bed capacity and provides services to over 1000 outpatients and inpatients on daily basis. A total of over 150 midwives provide maternal and child health services at the hospital. The hospital had an average monthly delivery of approximately 600 babies in 2019.

The Tema General Hospital is a 399-bed capacity hospital which accommodates approximately 500 patients daily. It has a midwifery staff strength of 90 and had an average of approximately 460 deliveries per month in 2019. Postnatal care at both health facilities is conducted according to national guidelines adapted from WHO recommendations for postnatal care. Mothers who delivered via spontaneous vaginal delivery were typically discharged after 24 hours in the absence of complications while mothers who delivered by caesarian section were discharge averagely between three to 5 days after surgery. Both hospitals have postnatal clinics which provide PNC to mothers and their babies at 1–2 weeks and 6 weeks.

### Study population

The study included mother-singleton baby pairs delivered in the Greater Accra Regional and Tema General hospitals during the study period.

### Eligibility criteria

Mother-baby pairs were eligible for inclusion if mother was at least 18 years of age and have access to a functional mobile phone, discharged from hospital but not have left the hospital at time of recruitment, not involved in another study and had no intention of moving out of the Greater Accra Region within 3 months after delivery. Exclusion criteria were babies born preterm, babies with major congenital anomaly or admitted to the neonatal intensive care unit (NICU) after delivery for severe illness and babies with either or both parents being a health worker.

### Sample size

Based on prior data from an initial pilot study conducted at the two hospitals that showed that the proportion of infant illness among mother-baby pairs in the usual PNC alone arm was 25% and our hypothesis that the risk of childhood illness in the telephone-based PNC relative to the usual PNC alone is reduced by 50%, with a two-sided type I error rate of 5, 80% power and 1:1 allocation ratio, a sample size of 186 was calculated for each arm given an anticipated loss to follow-up rate of 27% observed in a neonatal follow-up study [22]. A total of 200 mother-baby pairs per study arm were studied. The sample size was proportionately allocated to the hospitals based on the number of deliveries per month.

### Intervention

The intervention tested was telephone-based PNC delivered to mother-baby pairs in the intervention arm in two parts:

1. Part one: Nurse-led telephone-based health education and support for mother within 48 hours after discharge from hospital
2. Part two: Access of mother to a midwife on-call during the first 6 weeks after delivery (postnatal period) to ask questions about their own or baby’s health and care, when the need arose

This intervention was given to mother-baby pairs in addition to the usual PNC services provided by the health facility.

Content of telephone call to the mother was based on a client education protocol validated and used in Ecuador [18] and adapted to reflect the National Safe Motherhood Service Protocol as well as WHO and UNICEF guidance on care of the newborn [8, 23] (Table 1). The telephone-based education focused on educating mothers on key maternal and newborn care issues. Mother-baby pairs in the control arm received only the usual health facility-based PNC services.

**Table 1:** Content of postnatal education session

Topic	Discussion Points
**Newborn**	
Cord and skin care	Use of methylated spirit or Chlorhexidine for umbilical cord care; (no application of traditional substances), Keep baby warm (skin-to-skin contact)
Feeding	Exclusive breastfeeding, Techniques: Positioning and latching, Frequency of feeding (on demand), wake to feed, burp after each feed; no juice, water, honey, or solids; monitor output (check diaper)
Safe sleeping	Sleep on back; swaddle tightly; no loose blankets or stuffed animals; avoid co-sleeping if possible
Environment	No smoke; quiet environment; supervise other children with newborn
Danger signs	
Severe jaundice	Seek care immediately
Excessive crying	Check for danger signs; change diaper; swaddle; burp; help mother identify sources of support
Scheduled postnatal appointments	2-week and 6-week post-partum; vaccination schedule; weight check; birth registration
**Mother**	
Bleeding	Bleeding for 2–6 weeks is normal; it decreases with time; seek medical attention for heavy bleeding (> 1 pad/h) or fainting
Pain	Paracetamol or Diclofenac suppository: seek medical attention if not well controlled
Voiding	Avoiding constipation; seek medical attention if painful voiding or incontinence
Contraception	No intercourse for 6 weeks (after lochia has stopped and perineal wounds healed); review types and effectiveness of contraception
Infection recognition and prevention	Fever; pain with urination; bleeding/red/painful breasts; proper wound and breast care; personal hygiene including hand washing with soap under running water, care of the genital area (keep clean and dry, change perineal pads 4–6 hourly in first week and then twice daily, no douching, loose fitting clothing)
Mood	Postpartum depression is common; signs of postpartum depression; seek medical attention if experience depression, anxiety, suicidal; periodic rest during day when baby is sleeping

Adapted from Maslowsky et al., 2018

### Outcome measures

The primary outcome of the study was proportion of infants who fell ill within the first 3 months after delivery. Proportion of mothers who fell ill within the first 3 months after delivery was co-primary outcome. Secondary outcomes of proportion of mothers practicing exclusive breastfeeding, using contraceptives and those who had postpartum depression were also measured but not reported in this paper. The outcome measures were assessed verbally through interview of the mothers at 3 months after delivery.

Infant illness was defined as any acute health problem of the child since birth requiring a visit to a clinic, chemical shop, pharmacy or for which medical help was sought for the child. Maternal illness was any acute health problem of the mother since discharge from the hospital requiring a visit to a clinic, chemical shop, pharmacy or for which mother sought medical help.

### Randomization

Each mother-baby pair was randomized into either of two study arms using block randomization technique. The randomization scheme was made up of computer-generated random numbers of 100 blocks and 4 participants per block by using web-based randomization software [24].

### Allocation concealment

The random allocation sequence generated was printed and concealed by masking tape from the research team enrolling the participants with access restricted only to independent assistants at the two hospitals who were not directly involved in the data collection. The sequence code was only revealed serially by these independent assistants by peeling off the masking tape on a case-by-case basis after a participant consented and was enrolled in the study.

### Blinding

The study was conducted as an open-label trial. Owing to the nature of the study, it was not possible to blind study participants (mothers) and investigators to the intervention received. However, research assistants who assessed the outcomes at the end of the follow-up period were not aware of the study groups to which the participants were allocated, and the intervention given to each group.

### Data collection procedure

The study participants were enrolled soon after delivery or within few days after delivery whilst still in the hospital. The mother-baby pairs were provided with basic information on the study design and objectives for their interest. Mothers willing to be part of the study were screened for eligibility using the inclusion and exclusion criteria. Eligible participants were taken through informed written consent process. Mothers who consented, were enrolled, interviewed to obtain data on their sociodemographic characteristics, obstetric history and household assets. The allocation of the mother-baby pair to either arm of the study was done after consent, enrollment and obtaining all the baseline information. The independent assistant at each hospital who had access to the concealed allocation sequence peeled of the masking tape sequentially to reveal the next group assignment for each participant enrolled. Participants in the intervention arm were asked to provide information on what time of the day they preferred to be called.

Mother-baby pairs in both arms were followed up through the hospitals’ routine follow-up. In both groups, mother-baby pairs received routine PNC and education provided by health facilities without interference from the research team.

Outcomes of maternal and child illness were assessed at 3 months. The mother-baby pairs were followed up while at home at 3 months and interviewed face-to-face on baby and maternal health using a structured questionnaire. They were called on phone to schedule the follow-up interviews. The interviews were arranged at a time and place convenient for the mothers. In instances where telephone interviews were preferred to the face-to-face interviews, the interviews were arranged at time convenient for the mothers. The interviews were initially planned to be conducted face-to-face but were not possible for all the participants. This was because the study period coincided with the initial peak phase of COVID-19 in the region and there were restrictions on movement and stay at home orders coupled with high level of uncertainty among the populace. Some participants were reluctant to have face-to-face meetings with the research assistants.

Outcomes were assessed for mother-baby pairs in the intervention and the control groups at 3 months after delivery. At each contact, the mother was asked if she or her baby had been ill since the last physical contact with the research team, and if so, when the illness started and ended. Other data collected were whether care was sought for illness and from where. The mothers were provided counselling and support when needed during the outcome assessment period. Participants were defined as lost-to-follow-up when they were unable to be reached within 1 week after the 3 months follow-up.

### Quality control

The research assistants were trained on the data collection process and the delivery of the intervention. The questionnaires were pretested to ensure clarity and ambiguities resolved. The data were entered directly into the Open Data Kit (ODK) software downloaded onto portable mobile devices to prevent errors. The ODK database was reviewed daily to ensure that the questionnaires were properly filled. In order to ensure that the outcome assessors were not aware of the arm of the study to which participants randomized, different set of research assistants made up of community health nurses were used as outcome assessors. The telephone call conversations during the intervention delivery were audio recorded if permission was granted by the participants and reviewed to ensure the intervention was delivered as planned.

### Data management and analysis

Data from the field were directly entered into ODK, exported to Microsoft Excel and then to STATA Version 16.0 [25] for analysis. Data was cleaned by running frequencies of all variables that were used in the analysis. Incorrect data points were resolved by contacting the study participants and appropriate corrections done. Descriptive statistics were used to describe baseline socio-demographic and obstetrics characteristics as well as distribution of infant and maternal illness among participants in the study arms.

Wealth index was constructed using the principal component analysis (PCA), as a proxy of the SES of the household of each mother-baby pair using housing characteristics and utility variables. The items included in the wealth index calculation were: household ownership of assets (clock, mosquito net, bed, livestock, blender, table, room divider, fridge, fan, sewing machine, washing machine, computer, air conditioner, mobile phone, radio set, television, bicycle, motor bike, car, land,), characteristics of materials used for housing (material used for roof, floor and wall of house, number of rooms) and access to basic services (electricity supply, source of water, access to toilet facility, type of fuel used). Based on the wealth scores, households were put into five categories of wealth: quintile 1, quintile 2, quintile 3, quintile 4 and quintile 5.

In order to cater for the effects of missing data, minimize bias, preserve the sample size and representativeness of the data, multiple imputation (MI) was used to impute missing outcome data for analysis by ITT. The ITT method of analysis was used for all participants based on how they were originally randomized. The imputation model predicting missing outcome values was implemented by the multiple chain equations method with the assumption that missing outcome data were missing at random. The multiple imputed data was used for the ITT analysis. Proportions of mothers and infants who fell ill were compared between the study arms using the Chi-squared-test with its resultant *p*-values. Risk ratios (RR) were calculated to determine the strength of association between exposures and outcomes. Statistical evidence of associations between the intervention and each outcome was assessed using 95% confidence interval (CI). In order to examine whether there was effect heterogeneity across subgroups, post hoc subgroup analyses were done for the infant illness and maternal illness to explore any differences in magnitude of intervention effect among different categories of participants. The categories considered were highest level of education, antenatal care attendance, parity, and socioeconomic status. With an initial significance level of 0.05 and four post hoc subgroup analyses done, adjustments for multiplicity were made using the Bonferroni correction. The significance level was set at 0.01 for all the subgroup analyses.

Subgroup analyses was done using modified Poisson regression model with robust error variances. The interaction *p*-values were used to determine any significant subgroup effects.

To investigate the effect of protocol violation on the results obtained in the primary analysis, PP analysis was done by restricting analysis to only participants who complied with the protocol (were reached on phone within 48 hours after discharge and taken through the postnatal education according to the checklist). The sensitivity analysis was done by comparing the ITT and PP analyses.

This trial was retrospectively registered on 09/04/2021 with the International Standard Randomized Controlled Trial Number (ISRCTN) Registry with number ISRCTN46905855.

## Results

**Fig. 2:**
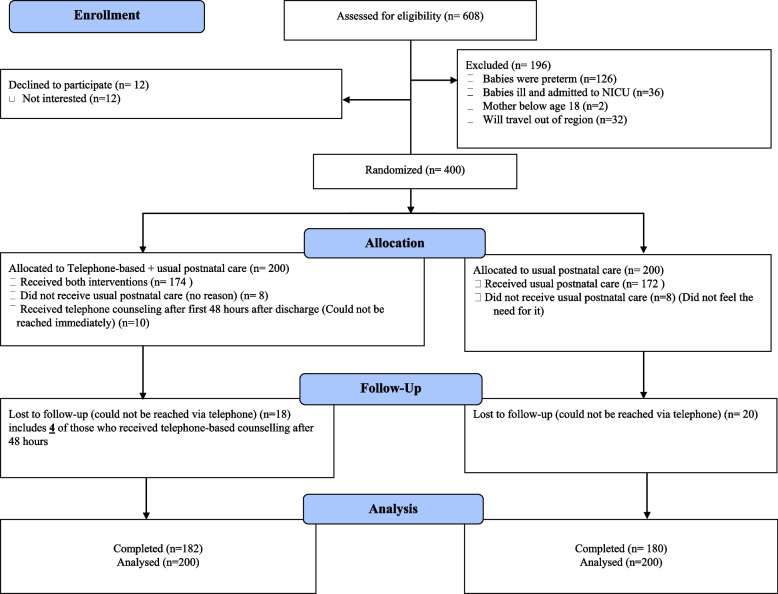
CONSORT 2010 Flow Diagram for progression of participants through the trial

A total of 608 mother-baby pairs were assessed for eligibility, and those eligible were enrolled and studied. Of these, 196 (32.2%) were found to be ineligible and were excluded from the study. Of the remaining 412 eligible participants, 12 declined to participate because they were not interested. The remaining 400 participants were randomized to receive either the usual PNC or telephone-based PNC. Of the 400 participants randomized, 38 (9.5%) were lost to follow-up. In the telephone-based arm, 10 (5.0%) mothers did not receive telephone-based counselling within the planned 48 hours because they could not be reached via telephone. Of the 10 who could not be reached within 48 hours, six (60.0%) were reached after 48 hours and four (40.0%) were never reached and were included in the lost to follow-up. Eight mother-baby pairs from each the study arms did not receive the usual 2 and 6-week PNC because they did not feel the need to. The remaining 362 mother-baby pairs (182 from the telephone-based arm and 180 from the usual care arm) completed the study (Fig. 2). The per-protocol analysis comprised 340 mother-baby pairs: 168 mother-baby pairs in the telephone-based arm and 172 mother-baby pairs in the usual PNC arm whilst the ITT population was based on mother-baby pairs as they were randomized.

### Baseline sociodemographic characteristics of trial participants

The mean age of all mothers in the study population was 30.1 (+/− 5.8). Mothers in the telephone-based arm had a mean age of 29.9 (+/− 5.5) years and those in the usual PNC arm had a mean age of 30.3 (+/− 6.0) years. Of the 400 mothers, 321(80.3%) were married at the time of the study (Table 2).

**Table 2:** Sociodemographic characteristics of study participants, Greater Accra Regional and Tema General Hospitals, 2020

Characteristics	Total (***N*** = 400) n (%)	Intervention (***N*** = 200) n (%)	Control (***N*** = 200) n (%)
Mean age (years)	30.09 (5.8)	29.90 (5.5)	30.29 (6.0)
Mean number of children (including current child)	1.49 (1.5)	1.44 (1.4)	1.54 (1.5)
**Marital Status**			
Never Married	23 (5.8)	10 (5.0)	13 (6.5)
Currently Married	321 (80.3)	162 (81.0)	159 (79.5)
Cohabiting	56 (14.0)	28 (14.0)	28 (14.0)
**Highest Level of education**			
None	18 (4.9)	9 (4.8)	9 (5.0)
Primary	135 (36.7)	67 (36.0)	68 (37.4)
Secondary	141 (38.3)	75 (40.3)	66 (36.3)
Tertiary	74 (20.1)	35 (18.8)	39 (21.4)
**Occupation**			
Employed (Government)	38 (9.5)	15 (7.5)	23 (11.5)
Employed (Private)	26 (6.5)	15 (7.5)	11 (5.5)
Self employed	97 (24.3)	44 (22.0)	53 (26.5)
Trader	142 (35.5)	73 (36.5)	69 (34.5)
Unemployed	56 (14.0)	25 (12.5)	31 (15.5)
Others	41 (10.3)	28 (14.0)	13 (6.5)
**Religion**			
Christian	339 (84.8)	165 (82.5)	174 (87.0)
Muslim	61 (15.3)	35 (17.5)	26 (13.0)
**Ethinicity**			
Akan	208 (52.0)	98 (49.0)	110 (55.0)
Ga	44 (11.0)	22 (11.0)	22 (11.0)
Ga-Adangme	7 (1.8)	1 (0.5)	6 (3.0)
Ewe	72 (18.0)	39 (19.5)	33 (16.5)
Krobo	5 (1.3)	3 (1.5)	2 (1.0)
Others	64 (16.0)	37 (18.5)	27 (13.5)
**Child ever died**			
Yes	26 (6.5)	12 (6.0)	14 (7.0)
No	374 (93.5)	188 (94.0)	186 (93.0)
**Possession of NHIS**			
Yes	390 (97.5)	195 (97.5)	195 (97.5)
No	10 (2.5)	5 (2.5)	5 (2.50)
**Socioeconomic Status**			
First Quintile	34 (8.5)	17 (8.5)	17 (8.5)
Second Quintile	34 (8.5)	15 (7.5)	19 (9.5)
Third Quintile	33 (8.3)	18 (9.0)	15 (7.5)
Fourth Quintile	35 (8.8)	20 (10.0)	15 (7.5)
Fifth Quintile	264 (66.0)	130 (65.0)	134 (67.0)

### Baseline obstetric characteristics of study participants

Most 386 (96.5%) of the mothers had attended antenatal clinic at least four times for the index pregnancy; 193 (96.5%) each from the telephone-based and the usual PNC arms of the study. Mothers delivering their index babies via vaginal delivery were 157(39.3%).

For 102 (25.5%) of the 400 mothers included in the study, the index pregnancy was their first pregnancy whilst 57 (14.3%) of them had given birth at least four times prior to the delivering of their current baby. The mean birth weight of the babies delivered was 3.3 (+/− 0.5) kg with the mean birth weight of babies in the telephone-based and usual PNC arms being 3.3 (+/− 0.5) kg and 3.3 (+/− 0.5) kg respectively (Table 3).

**Table 3:** Obstetrics characteristics of the study participants, Greater Accra Regional and Tema General Hospitals, 2020

Characteristics	Total (***n*** = 400)	Intervention (***n*** = 200)	Control (***n*** = 200)
**Number of antenatal clinic attendance**			
1	6 (1.5)	4 (2.0)	2 (1.0)
2	2 (0.5)	2 (1.0)	0 (0.0)
3	6 (1.5)	1 (0.5)	5 (2.5)
≥ 4	386 (96.5)	193 (96.5)	193 (96.5)
**Mode of delivery**			
Vaginal Delivery	157 (39.3)	85 (42.5)	72 (36.0)
Caesarian Section	243 (60.8)	115 (57.5)	128 (64.0)
**Number of previous pregnancies**			
1	102 (25.5)	57 (28.5)	45 (22.5)
2	85 (21.3)	39 (19.5)	46 (23.0)
3	103 (25.8)	50 (25.0)	53 (26.5)
≥ 4	110 (27.5)	54 (27.0)	56 (28.0)
**Number of full-term births**			
0	52 (13.0)	27 (13.5)	25 (12.5)
1	113 (28.3)	58 (29.0)	55 (27.5)
2	97 (24.3)	47 (23.5)	50 (25.0)
3	81 (20.3)	43 (21.5)	38 (19.0)
≥ 4	57 (14.3)	25 (12.5)	32 (16.0)
**Sex of baby**			
Male	213 (53.3)	99 (49.5)	114 (57.0)
Female	187 (46.8)	101 (50.5)	86 (43.0)
**Apgar Scores**			
At one minute	6.69 (0.9)	6.68 (1.0)	6.71 (0.8)
At five minutes	8.27 (0.9)	8.27 (0.9)	8.28 (0.8)
**Birth weight**	3.28 (0.5)	3.30 (0.5)	3.25 (0.5)
**Gestational age at delivery**	39.00 (1.2)	38.99 (1.1)	39.01 (1.3)

### Distribution of infant and maternal health illness

Of the 362 participants who completed the study, 257 (71.0%) of the infants were reported to have fallen ill at one point in time during the 3 months of follow-up while 119 (32.9%) mothers fell ill at least once during the same period (Table 4).

**Table 4:** Distribution of maternal and infant health illness, among the study participants after three months of follow-up, Greater Accra Regional and Tema General Hospitals, 2020

Outcome	Total ***n*** = 362	Intervention ***n*** = 182	Control ***n*** = 180	X^**2**^ Statistic	***p***-value
**Infant health status**				10.84	0.001
Ill	257 (71.0)	115 (63.2)	142 (78.9)		
Not ill	105 (29.0)	67 (36.8)	38 (21.1)		
**Maternal health status**				5.87	0.02
Ill	119 (32.9)	49 (26.9)	70 (38.9)		
Not ill	243 (67.1)	133 (73.1)	110 (61.1)		

### Risk of infant and maternal illness

For the outcome of infant illness, 62.5% infants in the telephone-based arm and 77.5% of infants in the usual PNC arm fell ill at least once during the follow-up period. The risk of falling ill was 20% less in the telephone-based arm than the usual PNC arm in both the ITT analysis [RR = 0.8 (95%CI = 0.71–0.92] and the PP analysis [RR = 0.8 (95%CI = 0.67–0.89)] (Table 5). For maternal illness as outcome, 27.5% of mothers in the telephone-based arm compared to 38.5% of mothers in the usual PNC group fell ill at one point in time during the three-month follow-up period. The risk of a mother falling ill was 30% lower in the telephone-based arm than that in the usual PNC arm in the ITT population analysis [RR = 0.7 (95%CI = 0.54–0.95)] and the PP group [RR = 0.7 (95%CI = 0.51–0.94)] (Table 5).

**Table 5:** Intention-to-treat and per-protocol analysis of comparison of infant and maternal illness between study arms, Greater Accra Regional and Tema General Hospitals, 2020

Outcome	Intervention % (95% CI)	Control % (95% CI)	RR (95% CI)
**ITT Population Analysis (*n*** **= 400)**			
Infant illness	**62.5 (55.39–69.23)**	**77.5 (71.08–83.09)**	**0.8 (0.71–0.92)**
Maternal illness	**27.5 (21.44–34.24)**	**38.5 (31.72–45.62)**	**0.7 (0.54–0.95)**
**PP Population Analysis (*n*** **= 340)**			
Infant illness	**60.7 (52.90–68.15)**	**78.5 (71.59–84.38)**	**0.8 (0.67–0.89)**
Maternal illness	**28.0 (21.34–35.41)**	**40.1 (32.73–47.85)**	**0.7 (0.51–0.94)**

**Fig. 3:**
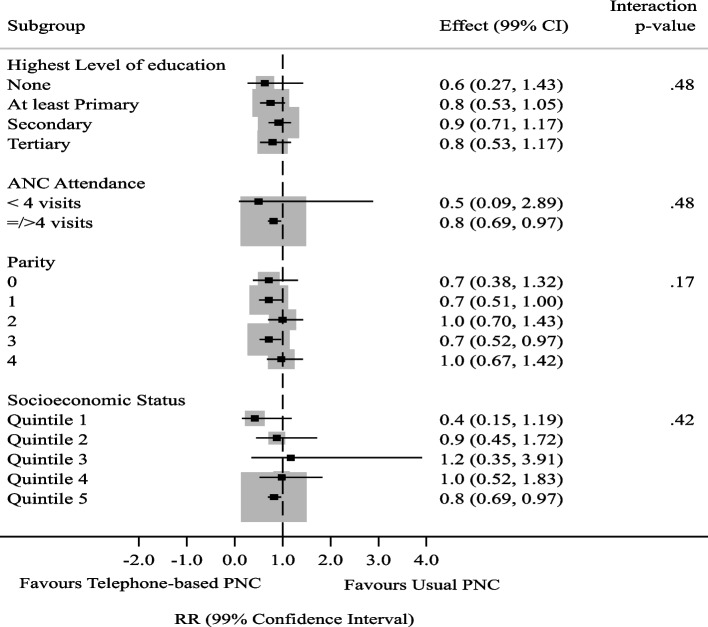
Subgroup Analysis for Infant Illness by Educational Level, ANC attendance, Parity, Mode of delivery, Socioeconomic status

For subgroup analysis based on educational level, ANC attendance, parity and socioeconomic status, there was no subgroup effect (Fig. 3). There was homogeneity of effect of risk reduction by the intervention across all the subgroups.

**Fig. 4:**
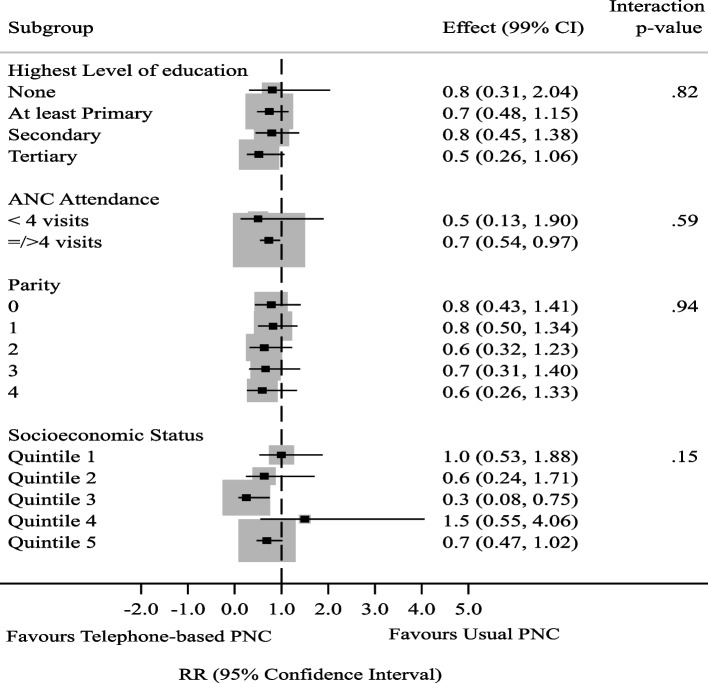
Subgroup Analysis for Maternal Illness by Educational Level, Parity, Mode of delivery, Socioeconomic status

The results of the post-hoc subgroup analyses for highest educational level, ANC attendance, parity and socioeconomic status indicate consistency of effects. All the categories maintained similar pattern of significant reduction in risk of mothers falling ill in the intervention compared to the risk of mothers falling ill in the control group (Fig. 4).

### Acceptability of intervention

Most 168 (92.3%) of mothers in the intervention arm were either satisfied 99 (54.4%) or very satisfied 69 (37.9%) with respect to the intervention.

## Discussion

This study sought to assess the effectiveness of adjunct telephone-based postnatal PNC on health of infants and their mothers. The study found that compared to their counterparts who received the usual PNC, mother-baby pairs who received adjunct telephone-based PNC were at a much lower risk of falling ill within the first 3 months after delivery. This finding brings to the fore the importance of adoption of mobile health (mHealth) interventions in Ghana’s health care delivery. This is particularly important in the light new wave of telemedicine in Ghana heightened by the ongoing COVID-19 pandemic [26]. In instances in the maternal, newborn and child health continuum of care, where virtual patient care, involving remote exchange of health information between health provider and patient becomes desirable either as a complementary or sole intervention, telephone-based intervention may serve as a foundation.

This study showed that complementing the usual PNC with telephone follow-up significantly reduces the likelihood of illness in both mothers and their babies within 3 months of follow-up. Infants in the intervention arm were significantly less likely than those in the control arm to have fallen ill at least once within the 3-month follow-up period. This indicates that when combined with the usual PNC, telephone-based postnatal follow-up is an effective intervention to reducing infant and maternal morbidity and its attendant consequences of increased expenditure on health as well as overstretched health services. This finding is congruent with findings from a number of other studies conducted in Ecuador and United States of America where significantly fewer infant morbidities were recorded among infants in the telephone-based intervention arm compared to the those in the control arm [18, 19].

This finding is considered an important step towards reducing infant mortality. This is because neonatal mortality remains the main driver of child death globally. Nearly half of all under five deaths (approximately 41%) occur among babies in the neonatal period [27]. Currently in Ghana, whilst infant and under five mortality rates have progressively declined to 21% and 31% respectively from 1990 to 2014, reduction in neonatal deaths has virtually stagnated, with a much slower decline rate of 3% (from 30 to 29 deaths per 1000 live births) [28]. Neonatal mortality thus remains a major contributor to overall mortality of children less than 5 years. Reducing acute illness in the neonatal period is therefore critical in reducing overall child mortality.

While telephone-based PNC significantly reduced the proportion of infant and maternal illness, the effect of the intervention did not vary between subgroups. This finding of consistency of intervention effect across trial subgroups has important implications for practice as decisions on implementation of the intervention will not require considerations of individual characteristics. However, this conclusion has to be given a careful consideration bearing in mind that failure to find heterogeneity across subgroups does not imply definitely that intervention effect seen overall applies to all participants regardless of peculiar characteristics [29]. The lack of subgroup effect seen in this study may be a false negative finding as the study was not powered to determine subgroup effect [30].

The intervention was readily acceptable by the mothers in the intervention arm as evident in their overwhelming expression of satisfaction. Similar findings were observed in Ecuador where almost all the mothers in the telephone intervention arm agreed that the intervention had a positive impact on their lives and they would like to benefit from it in future pregnancies [18]. This finding is however not uncommon as telephone-based interventions in healthcare have received positive feedback from patients and caregivers in many settings [31–33]. This is probably because of the tremendous flexibility and accessibility it offers. The high level of satisfaction observed in this study, coupled with Ghana’s high mobile phone penetration rate of over 130% [20] and national drive for mHealth [34] favour the intervention as a candidate for adoption and possible scale up as mainstream standard of practice for specific targeted populations.

As a novel strategy of PNC delivery in the Ghana Health Service, adoption and scale-up should be thoroughly thought through taking into consideration first the clinical importance of the effect. Also to be considered in plans for scale-up are availability of human, material and financial resources required to drive the scale-up [35]. Adequate provision of the human and material resources including mobile phones and call credits are key sustainability measures should precede large-scale adoption. Formalizing this intervention has the potential to reduce undue burden on nurses who have been using their personal phones and call credits informally to support patient care [36, 37]. Although a simple intervention which made use of available human resources without additional special training requirements, a cost-effectiveness study is also warranted as a prerequisite for scale-up. Additional studies should also explore variants of the intervention which may either shift the cost of calling to mothers or provide opportunity for cost sharing.

Being an interventional study with a low attrition rate, the study has a high internal validity. However, there are some limitations which must be considered in the interpretation of the results. Firstly, there was a possibility of the Rosenthal effect where outcome assessors might get to know the study arm to which the mothers’ belonged during the interview process and form perceptions which might influence the responses. This was minimized by using different set of research assistants blinded to the allocation intervention for the outcome assessment. Secondly, there was a risk of contamination where participants in the control arm might receive some information from their counterparts in the intervention arm. This was possible because participants interacted among themselves and exchanged telephone contacts while on admission at the hospitals. This risk of contamination in this study was however low as mothers are unlikely to deliver the health education and provide support as trained health workers who are experienced in providing health care. In addition, health workers and their spouses were excluded to minimize co-intervention bias where participants may have received other interventions be it formal or informal which is likely to have contaminated the study. Data on materials used for housing were obtained from interviews rather than observation as the mothers were enrolled at the health facility rather than their homes. Furthermore, both face-to-face and telephone interviews were used to assess outcomes. Though the potential for social desirability bias and measurement errors exists for using this mixed-mode data collection techniques, these are unlikely to affect conclusions drawn from the study as no single mode for outcome assessment was limited to participants in a particular arm of the study. The mixed-mode data collection approach on the other hand, was used to improve response rate and thus reduce the loss to follow-up rate. Finally, being an RCT, the external validity is limited by the urban setting of the trial, the category of health facilities used and the eligibility criteria. Generalizability of this intervention to non-urban settings as well as mothers with characteristics not meeting the eligibility criteria, cannot be guaranteed. Despite these limitations, the study provides potentially useful information that can be reasonably considered for application to targeted populations.

## Conclusion

Telephone-based PNC consisting of providing postnatal health education and support beginning 48 hours after discharge plus granting a nursing mother telephone access to a dedicated health worker during the postnatal period when implemented in addition to the usual PNC resulted in statistically significant reduction in proportion of mothers and their babies who fall ill during the first 3 months postpartum. The intervention was largely acceptable to the study participants and shows promise for consideration as an adjunct to usual PNC to improving infant and maternal health outcomes in some settings. Future research is however needed to determine the clinical importance of this finding, its cost effectiveness as well as the optimum format, timing, frequency, and duration of telephone follow-up intervention required to be considered as reasonable option for PNC.

## Data Availability

The datasets used and/or analysed during the current study are available from the corresponding author on reasonable request.
